# Radiofrequency Ablation for Recurrent Pleural Mesothelioma

**DOI:** 10.3390/cancers18030381

**Published:** 2026-01-26

**Authors:** Hiroshi Kodama, Kozo Kuribayashi, Haruyuki Takaki, Kosuke Matsuda, Takashi Shinkai, Reona Wada, Atsushi Ogasawara, Masaki Hashimoto, Daichi Fujimoto, Toshiyuki Minami, Soichiro Funaki, Takashi Kijima, Koichiro Yamakado

**Affiliations:** 1 Department of Radiology, Hyogo Medical University, 1-1 Mukogawa-cho, Nishinomiya 663-8501, Hyogo, Japan; 2 Department of Respiratory Medicine and Hematology, Hyogo Medical University, Mukogawa-cho, Nishinomiya 663-8501, Hyogo, Japan; 3 Division of Thoracic Surgery, Department of Surgery, Hyogo Medical University, 1-1 Mukogawa-cho, Nishinomiya 663-8501, Hyogo, Japan

**Keywords:** pleural mesothelioma, radiofrequency ablation, safety, clinical outcomes, survival

## Abstract

**Background/Objectives**: Pleural mesothelioma (PM) frequently recurs despite multimodal therapy. Here, we aimed to retrospectively evaluate the safety and potential clinical benefit of radiofrequency ablation (RFA) for recurrent PM. **Methods**: Fourteen consecutive patients underwent CT-guided RFA between July 2019 and June 2025. The cohort comprised 13 men and 1 woman, with a median age of 69 (range, 54–77) years. All patients had previously received systemic therapy, and 12 had undergone surgery. Seven patients (50%) presented with multiple lesions, and 25 tumors (median diameter 1.8 cm; range, 0.5–7.0 cm) were treated in 23 sessions. Outcomes assessed were local tumor control, complications, and survival. Local progression and overall survival were estimated using Kaplan–Meier analysis. Adverse events were classified according to the Society of Interventional Radiology guidelines. **Results**: Technical success was achieved in all sessions. Two tumors showed local recurrence, corresponding to 1- and 2-year local progression rates of 10.6%. Seven patients showed distant metastases, most of whom subsequently received systemic therapy. Three patients died, two from disease progression and one from treatment-related gastrointestinal perforation during therapy for an unrelated cancer. The overall survival rates were 100%, 100%, and 60% at 1, 3, and 5 years, respectively. Major and minor complications occurred in one case each (4.3%): a refractory skin ulcer and retroperitoneal hematoma, respectively. **Conclusions**: RFA was technically feasible and generally well tolerated and helped achieve encouraging local control and survival in patients with recurrent PM, warranting further evaluation of RFA as a complementary approach in multimodal treatment strategies.

## Simple Summary

Pleural mesothelioma is a rare but highly aggressive malignancy that often relapses, posing major therapeutic challenges. We investigated radiofrequency ablation (RFA) as a minimally invasive option for patients with recurrent disease. We retrospectively reviewed the data of 14 patients treated with RFA to assess its safety and clinical impact. The procedure was feasible in all sessions, with only one major complication (4.3%) and one minor event (4.3%). Local tumor control was achieved in 92% of treated lesions, and the estimated overall survival at 3 and 5 years reached 100% and 60%, respectively. These findings indicate that RFA may be a valuable addition to current treatment strategies for recurrent pleural mesothelioma. Prospective studies are warranted to confirm our findings and clarify the integration of RFA into multimodal care.

## 1. Introduction

Pleural mesothelioma (PM) is an uncommon but highly aggressive malignancy associated with poor survival outcomes [1]. A universally accepted treatment strategy has yet to be established, and prognosis remains dismal despite the use of multimodal approaches. Even with combinations of surgery, systemic chemotherapy, and radiotherapy, the 5-year overall survival rarely exceeds 10% [1,2]. Moreover, the role of surgery itself has become increasingly controversial. A recent phase III randomized controlled trial demonstrated that median overall survival was not improved with surgical intervention; instead, survival was slightly shorter than with chemotherapy alone, and serious adverse events occurred significantly more frequently in the surgical arm [3]. These findings underscore the limitations and morbidity associated with aggressive surgical approaches in PM.

Systemic therapy has undergone substantial evolution in the past decade. Immune checkpoint inhibitors (ICIs), particularly nivolumab and ipilimumab, have extended median survival from approximately 12 months to nearly 18 months [4]. While these results represent important progress, ICIs are also associated with a considerable rate of immune-related toxicities. More than one-fifth of patients discontinue treatment due to adverse events, highlighting the difficulties of sustaining long-term systemic therapy in this population [4,5,6]. Radiation therapy has also advanced, particularly with the introduction of intensity-modulated radiotherapy (IMRT). Although small-cohort studies have suggested improved median survival times of up to 24 months [7,8], IMRT following radical surgery often results in substantial declines in pulmonary function, including approximately 30% reductions in forced vital capacity, forced expiratory volume in 1 s, and lung diffusing capacity [9]. These functional declines have significant consequences for quality of life in a patient population already burdened by disease-related respiratory compromise.

Despite these therapeutic advances, recurrence continues to be a major therapeutic obstacle, with reported rates ranging from 26% to 63% even after conventional multimodal treatment [10,11,12]. Recurrent PM is particularly difficult to manage because prior treatments often limit the feasibility of additional surgery or high-dose radiation, and systemic therapies may offer limited incremental benefit in the salvage setting. As a result, there is growing interest in identifying alternative, less invasive local treatments capable of providing meaningful tumor control while minimizing patient morbidity.

Radiofrequency ablation (RFA) is a minimally invasive technique that has demonstrated robust safety and efficacy across a wide range of tumors and anatomical sites [13,14,15,16]. Compared with surgery, RFA offers substantial advantages: it can be performed percutaneously, typically under conscious sedation and local anesthesia, and is associated with lower complication rates and shorter recovery periods. RFA is widely used worldwide, most notably for liver tumors—where local recurrence rates range from 2% to 41% [17]—and for primary and metastatic lung tumors, with recurrence rates of 8–21% [18]. Mortality associated with RFA remains below 1%, and major complication rates are generally around 10% [19,20].

Despite its broad applications, the role of RFA in pleural mesothelioma has been minimally explored. To date, only a single case report has described its use in PM [21], and no case-series studies have examined clinical outcomes systematically. Given the limited therapeutic options available for recurrent PM and the growing need for locally effective, minimally invasive treatments, RFA represents a potentially valuable strategy.

In the present study, we retrospectively analyzed outcomes of patients with recurrent PM treated with percutaneous RFA. Our objectives were to evaluate the safety profile of RFA, assess its efficacy in achieving local tumor control, and explore its potential contribution to prolonging survival in this challenging patient population.

## 2. Materials and Methods

### 2.1. Study Design

This retrospective single-center study was conducted with approval from the institutional review board of Hyogo Medical University (approval number: 202209-042), in accordance with the tenets of the Declaration of Helsinki. A waiver of patient consent for study enrollment was granted by the review board, but written informed consent was obtained from all patients for the RFA procedure.

### 2.2. Patient Selection

From July 2019 to June 2025, 14 consecutive patients underwent Computed Tomography (CT)-guided RFA for recurrent PM and were included in the analysis (Table 1). The cohort consisted of 13 men (92.9%) and 1 woman (7.1%), with a median age of 69 (range, 54–77) years. Histological diagnoses were epithelioid in 11 patients (78.6%), biphasic in 2 (14.3%), and sarcomatoid in 1 (7.1%). All patients had a history of systemic therapy, and 12 patients (85.7%) had undergone surgical resection (EPP in 2 and P/D in 10). Four patients had also received RT. Recurrence was established using Fluorodeoxyglucose Positron Emission Tomography/Computed Tomography (FDG-PET/CT) or based on progressive growth on serial CT in 9 patients (64.3%) and using biopsy in 5 patients (35.7%).

Exclusion criteria included patients with poor performance status (ECOG ≥ 3), severe comorbidities that contraindicated RFA, uncontrolled coagulopathy, or tumors deemed unsuitable for percutaneous ablation (e.g., lesions adjacent to major vessels). Patients who declined to provide informed consent for the procedure were also excluded.

Seven patients (50%) presented with multiple lesions (2–6 tumors). In total, 25 tumors were treated: 12 in the chest wall, 11 in the lung, and 1 in the liver. Tumor size ranged from 0.5 to 7.0 (median, 1.8) cm.

### 2.3. RFA Procedure

**Figure 1:**
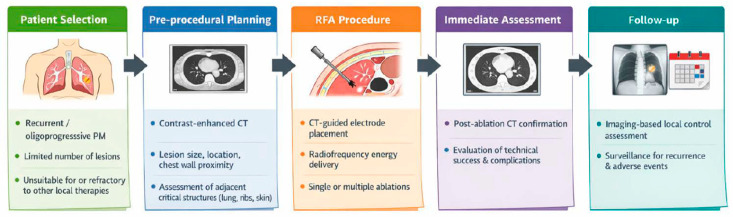
Schematic workflow of percutaneous RFA for recurrent PM.

To facilitate a clear and visual understanding of the clinical workflow of percutaneous RFA, a schematic illustration summarizing patient selection, pre-procedural planning, ablation procedures, and follow-up is provided (Figure 1). This schematic reflects the actual clinical practice described in Section 2.3, Section 2.4 and Section 2.5 and is intended to offer an educational overview without duplicating the technical details presented below.

A total of 23 RFA sessions were performed. One patient with a 7.0-cm tumor re-quired three sessions; all others were treated in a single session. Procedures were per-formed on an inpatient basis under local anesthesia and moderate sedation. Fentanyl citrate was administered for analgesia, and lidocaine was used for local anesthesia. Cefazolin was given prophylactically and continued for 2 days after treatment.

Real-time CT fluoroscopy (Aquilion One, Canon, Japan) guided the placement of 17- or 18-gauge internally cooled electrodes. Real-time CT fluoroscopy (Aquilion One, Canon, Otawara, Japan) guided the placement of 17- or 18-gauge internally cooled electrodes (Cool-Tip, Covidien, Boulder, CO, USA; or VIVA, STARmed, Gyeonggi, Republic of Korea). An 18-gauge electrode was preferentially used for lung lesions. The electrode number and exposure length were determined according to tumor size and morphology, with multiple electrodes used for tumors >3.0 cm. RF energy was delivered with an impedance-switching algorithm until three consecutive impedance spikes (≥30 W increase above baseline) occurred or a maximum of 12 min elapsed.

This schematic illustration provides an educational overview of the clinical workflow of percutaneous radiofrequency ablation (RFA), including patient selection, pre-procedural planning, CT-guided ablation, immediate post-procedural assessment, and follow-up. The figure reflects the actual clinical practice described in Section 2.3, Section 2.4 and Section 2.5 and is intended to facilitate visual understanding without duplicating technical details presented in the Methods.

### 2.4. Follow-Up

The patients underwent clinical examination, laboratory testing, and contrast-enhanced chest CT every 3 months. Follow-up continued until patient death or July 2025, whichever occurred first. The median follow-up duration was 22 (range, 2–72) months.

### 2.5. Endpoints and Definitions

Technical success was defined as complete tumor coverage within the ablation zone or disappearance of enhancement on CT within 1 week [15]. Adverse events (AEs) were classified according to the Society of Interventional Radiology guidelines [22]. Local tumor progression was defined as regrowth at the margin of an ablated lesion, whereas new lesions outside the ablation zone were categorized as distant metastases.

### 2.6. Statistical Analysis

Local progression and survival outcomes were estimated using the Kaplan–Meier method. Statistical analyses were performed with EZR software, version 1.61 (Saitama Medical Center, Jichi Medical University, Saitama, Japan), which is a graphical user interface for R (The R Foundation for Statistical Computing, Vienna, Austria; version 4.2.2). A *p*-value of <0.05 was considered statistically significant [23].

## 3. Results

### 3.1. Safety

Technical success was achieved in all 23 sessions (100%). No procedures were interrupted due to pain or intraprocedural complications. One patient developed a refractory skin ulcer, and another experienced a small subcutaneous hematoma not requiring intervention, corresponding to major and minor complication rates of 4.3% each.

### 3.2. Tumor Control and Recurrence

**Figure 2:**
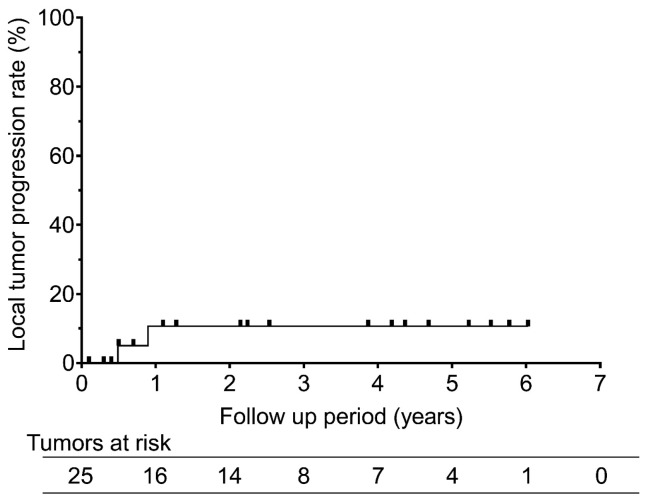
Local tumor progression rates after RFA for recurrent pleural mesothelioma. The graph shows the local tumor progression rates after radiofrequency ablation for recurrent pleural mesothelioma. The 1- and 2-year local overall survival rates were 10.6% and 10.6%, respectively.

At a median follow-up of 22 months, 7 of the 14 patients (50%) experienced recurrence: two patients showed both local and distant progression, and five showed distant metastases alone. The median time to progression was 11.7 (range, 5–20) months. Local progression occurred in 2 of the 25 treated tumors (8.0%), resulting in 1- and 2-year local progression rates of 10.6% (Figure 2).

After recurrence, 5 patients (71.4%) received systemic therapy (chemotherapy or immune checkpoint inhibitors), and 2 patients received best supportive care.

### 3.3. Survival Outcomes

**Figure 3:**
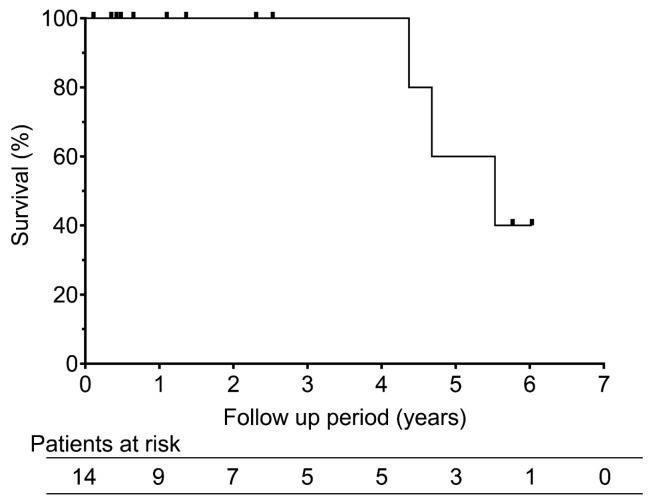
Overall survival rates after RFA for recurrent pleural mesothelioma. The graph shows the overall survival rates after radiofrequency ablation for recurrent pleural mesothelioma. The 1-, 3-, and 5-year overall survival rates were 100%, 100%, and 60%, respectively.

**Figure 4:**
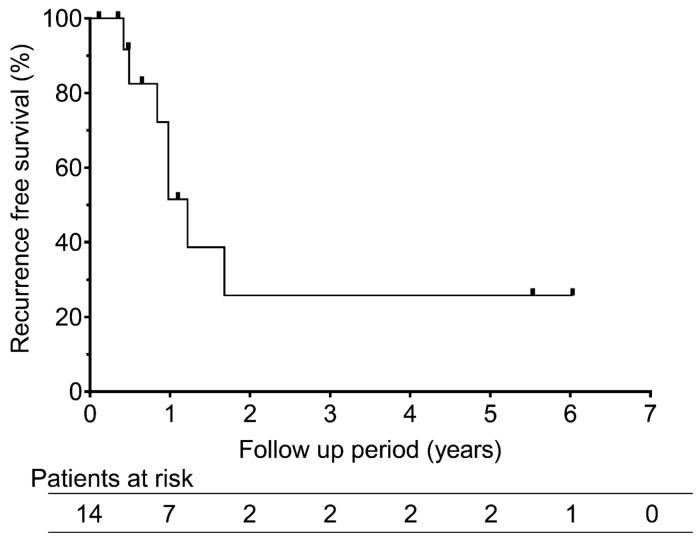
Recurrence-free survival rates after RFA for recurrent pleural mesothelioma. The graph shows recurrent free survival rates after radiofrequency ablation for recurrent pleural mesothelioma. The 1-, 3-, and 5-year recurrent free survival rates were 51.6%, 25.8%, and 25.8%, respectively.

Three patients (21.4%) died: two from tumor progression and one from gastrointestinal perforation during chemotherapy for esophageal carcinoma. The overall survival rates were 100% at 1 and 3 years and 60% at 5 years (Figure 3). The median overall survival duration was 5.5 years. Recurrence-free survival was 51.6% at 1 year and 25.8% at both 3 and 5 years, with a median of 1.2 years (Figure 4).

Because of the limited sample size, formal statistical analyses comparing survival outcomes according to prior surgical resection or previous radiotherapy were not feasible; therefore, these factors were evaluated descriptively.

## 4. Discussion

**Figure 5:**
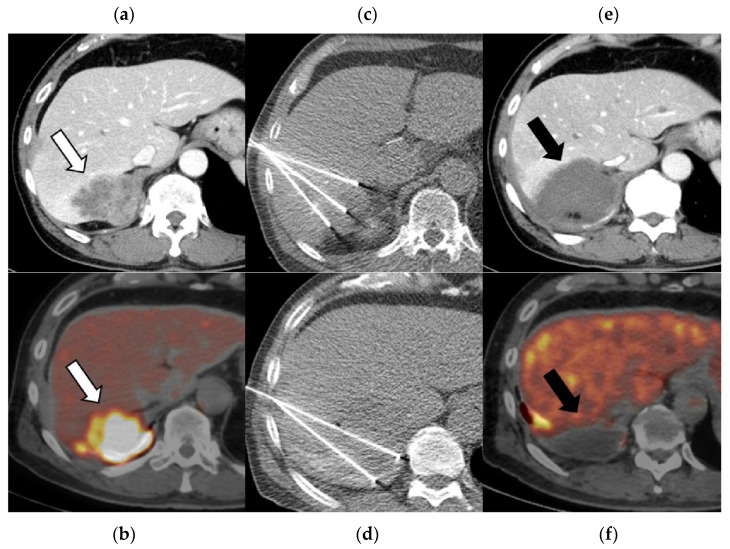
Images of 64-year-old man with sarcomatoid pleural mesothelioma. Baseline axial CT (**a**) and 18F-FDG PET/CT (**b**) images showing a large recurrent tumor invading the liver (white arrow). The patient underwent chemotherapy and immune checkpoint inhibitor therapy; however, the tumor continued to enlarge. Radiofrequency ablation (RFA) was subsequently performed in three sessions (**c**,**d**), resulting in successful tumor ablation. Tumor enhancement on contrast-enhanced CT and avid FDG uptake on PET/CT (black arrows) were no longer observed on follow-up CT (**e**) and PET/CT (**f**).

**Figure 6:**
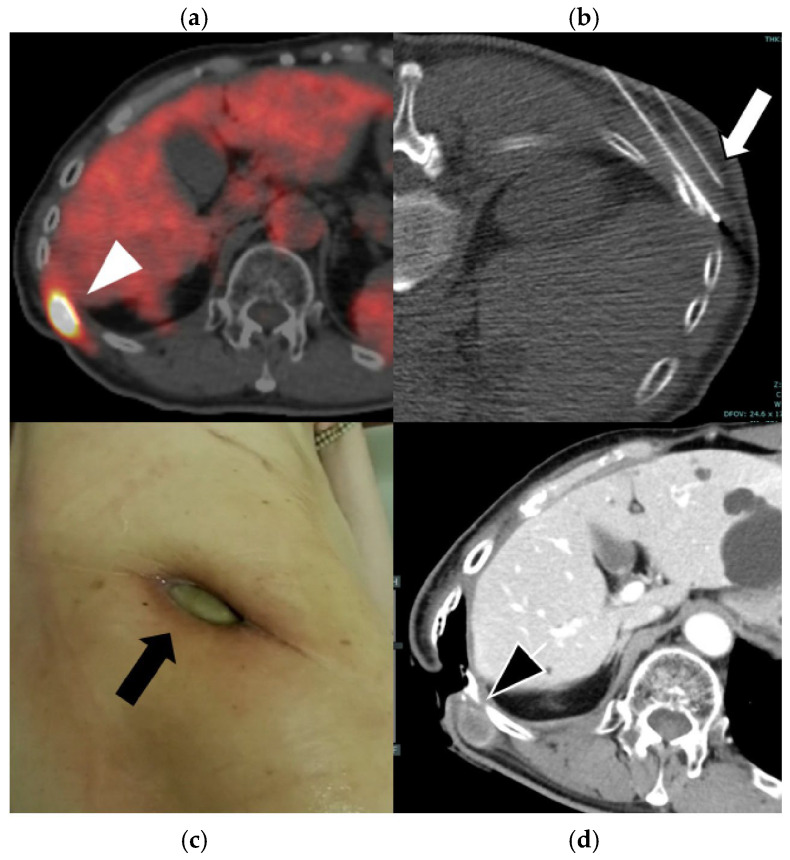
Images of a 60-year-old man with epithelioid pleural mesothelioma. Baseline axial 18F-FDG PET/CT image (**a**) showing focal avid FDG uptake in the right chest wall (white arrowhead). Despite undergoing multiple treatments, including surgery, radiation therapy, and systemic therapy, the recurrent tumor remained uncontrolled, and RFA was performed. The procedure was conducted with hydrodissection in the subcutaneous tissue (white arrow) and concurrent skin cooling using an ice pack to prevent thermal injury (**b**). However, a skin burn developed the following day, progressing to a refractory skin ulcer (black arrow) (**c**). Follow-up CT revealed local tumor progression (black arrowhead), but the patient declined further treatment (**d**).

This study demonstrated that percutaneous RFA for recurrent PM is technically feasible and associated with a low incidence of serious complications. The major AE rate of 4.3% was lower than that typically reported for lung RFA (10–30%) [24,25] and comparable to that for small chest wall series (<10%) [26,27]. Representative imaging findings are shown in Figure 5 and Figure 6. Nonetheless, the occurrence of a severe skin ulcer highlights the need for careful procedural planning and preventive measures when treating chest wall lesions.

Local tumor control was encouraging, with only 8% of treated tumors progressing, consistent with reported outcomes for lung RFA (10–20%) [25] and musculoskeletal ablation [28]. However, distant recurrence was frequent, occurring in half of the patients—a finding that aligns with published recurrence rates after surgery or systemic therapy (41–63%) [10,11]. These results reinforce the systemic nature of PM and the need to integrate local ablation with effective systemic therapy.

Radiation therapy remains a central component of multimodal PM management. IMRT, frequently employed after surgery, has demonstrated meaningful clinical benefit. Gomez et al. reported a median OS of 14.7 months following extrapleural pneumonectomy and IMRT [8], while Rimner et al. demonstrated median PFS and OS of 12.4 and 23.7 months, respectively, after chemotherapy and pleurectomy/decortication followed by IMRT [7]. Despite these advances, recurrence remains a major clinical challenge; a Japanese nationwide survey reported a 2-year local control rate of approximately 60% [16], emphasizing the unmet need for additional local modalities with durable disease control.

In this context, RFA may serve as a complementary or alternative option to radiotherapy. RFA is minimally invasive, repeatable, and preserves pulmonary function—an important advantage in previously irradiated or surgically treated patients. de Baère et al. demonstrated that radiofrequency ablation could be safely and repeatedly performed for lung tumors, reporting favorable midterm local control and survival outcomes in a single-center cohort, thereby supporting the feasibility of repeated RFA sessions in clinical practice [29]. The ability to deliver multiple ablations without cumulative pulmonary toxicity contrasts sharply with IMRT, for which repeated administration is often limited by lung function decline and radiation-induced fibrosis. Consistently, in the present study, RFA was used as a complementary local approach for recurrent pleural mesothelioma after multiple prior treatments, including systemic therapy, surgery, and radiotherapy. The acceptable safety profile and local control achieved in this context help to better define the potential therapeutic window of RFA as an additional modality within multimodal treatment strategies.

Evidence from other organs supports the applicability of RFA in PM. Local recurrence rates after RFA range from 2–41% in hepatocellular carcinoma [30] and 8–21% in lung tumors [25], with mortality rates generally <1% [14]. The 92% tumor control rate observed in the present study is comparable to, or slightly more favorable than, outcomes in these organs, despite the additional technical challenges posed by pleural anatomy.

Emerging data also suggest that RFA may potentiate systemic immunotherapy. Preclinical studies indicate that thermal ablation induces immunogenic cell death, releases tumor antigens, and enhances dendritic cell activation, thereby priming the immune system and potentially augmenting the efficacy of immune checkpoint inhibitors (ICIs) [31]. As ICIs have become standard first-line therapy for unresectable PM, combining RFA with ICI therapy—particularly for oligoprogressive lesions—may provide additional therapeutic benefit. Although clinical data remain limited, studies in other malignancies (HCC, lung cancer) offer a compelling rationale for exploring this strategy in PM.

Similarly, multimodal local therapy integrating RFA with radiotherapy (IMRT or SBRT) warrants investigation. For tumors >3 cm or those close to critical structures, RFA alone may be insufficient; consolidative SBRT following incomplete ablation, or RFA applied after partial radiotherapy response, may enhance local control while minimizing toxicity. Because thermal ablation produces a sharply demarcated necrotic zone, combining RFA with IMRT could also reduce high-dose radiation volumes, offering a lung-sparing alternative for previously irradiated patients.

Future directions may include biomarker-guided selection between RFA and radiotherapy. Imaging biomarkers such as ADC values, contrast enhancement patterns, or FDG-PET metabolic activity could potentially predict thermal versus radiation sensitivity. Furthermore, genomic features associated with PM aggressiveness—such as BAP1 loss or CDKN2A deletion—may correlate with responsiveness to local ablative therapies, although such genomic data were not uniformly available in the present retrospective cohort. Prospective studies integrating imaging and genomic profiling could refine patient selection and optimize treatment planning.

The rarity of PM must be considered when interpreting the clinical impact of our findings. Although the cohort is small, this study represents the largest case series to date evaluating RFA specifically for recurrent PM. For rare malignancies, even limited case series can provide valuable evidence and shape future research directions. The high local control rate, favorable survival, and low toxicity profile observed in this study support the potential incorporation of RFA into the multidisciplinary management algorithm for recurrent PM. At the same time, although this study represents the largest case series to date focusing on RFA for recurrent PM, the sample size remains limited. As a result, formal statistical conclusions regarding differences in outcomes according to prior surgical resection or previous radiotherapy could not be established. These issues should therefore be explicitly acknowledged as important unanswered questions that must be addressed in future studies.

## 5. Limitations and Future Directions (Future Research Question (FRQ)-Based)

Nevertheless, several limitations must be acknowledged. The retrospective, single-institution design and small sample size may introduce selection bias. Follow-up duration varied, possibly leading to an underestimation of late recurrences. In addition, treatment efficacy may be reduced for larger tumors (>3 cm), suggesting a potential role for combination strategies incorporating radiotherapy, immune checkpoint inhibitors (ICIs), or surgery.

Beyond these limitations, several important future research questions (FRQs) arise from the present study and warrant further investigation:

FRQ1: Does the therapeutic efficacy of RFA differ according to prior surgical resection or previous radiotherapy, and do these factors influence clinical outcomes in patients with recurrent pleural mesothelioma?

FRQ2: Can RFA delay the initiation of subsequent systemic therapy in oligoprogressive pleural mesothelioma, as assessed by time to further systemic therapy?

FRQ3: How can genomic profiling, including BAP1 status, contribute to patient selection and optimization of local ablative therapies such as radiofrequency ablation in pleural mesothelioma?

Accordingly, future multicenter prospective studies are needed to validate our findings, standardize procedural techniques, evaluate combination regimens such as RFA–ICI or RFA–IMRT, and identify clinical, imaging, or molecular biomarkers predictive of optimal local therapy selection.

In summary, RFA appears to be a feasible, safe, and effective minimally invasive treatment for recurrent pleural mesothelioma, offering durable local tumor control and complementing established treatments such as radiotherapy and systemic immunotherapy. These findings warrant further investigation and contribute to expanding therapeutic possibilities in this challenging disease.

## 6. Conclusions

In conclusion, this study demonstrates that percutaneous radiofrequency ablation (RFA) is a safe, feasible, and effective local treatment option for patients with recurrent pleural mesothelioma, achieving high technical success and durable local tumor control with minimal morbidity. In the current therapeutic era, in which immune checkpoint inhibitors have substantially reshaped the treatment landscape of mesothelioma, RFA represents a minimally invasive and repeatable modality that may further expand therapeutic options, particularly for patients with limited alternatives.

Importantly, the present findings highlight several clinically relevant questions that warrant further investigation. These include whether the efficacy of RFA differs according to prior surgical resection or radiotherapy, whether RFA can meaningfully delay the initiation of subsequent systemic therapy in oligoprogressive disease, and how genomic profiling—such as BAP1 status—may contribute to optimal patient selection and treatment optimization. Addressing these future research questions will be essential to fully define the role of RFA within multidisciplinary treatment strategies.

Although the sample size of this study is inherently limited by the rarity of the disease, our results provide important preliminary evidence supporting the incorporation of RFA into multidisciplinary care for recurrent pleural mesothelioma. Larger, prospective, and multi-institutional studies are warranted to validate these findings and to establish evidence-based frameworks for integrating RFA with systemic immunotherapy, advanced radiotherapy techniques, and biomarker-driven treatment strategies, with the ultimate goal of improving outcomes in this challenging disease.

## Figures and Tables

**Table 1:** Patient characteristics.

No	Age (Years)	Sex	Histological Subtype	Stage at Initial Treatment	Prior Treatment	Tumor Location	Max Tumor Diameter (cm)	Treated Tumor Number
1	55	M	Biphasic	T3N1M0	EPP, chemotherapy, Radiation	Chest wall	2.5	2
2	73	M	Epithelioid	T2N0M0	P/D, chemotherapy, ICI	Chest wall	2.3	2
3	64	M	Sarcomatoid	T4N0M0	Chemotherapy, ICI	Liver	7.0	1
4	54	M	Epithelioid	T2N0M0	P/D, chemotherapy, ICI, Radiation	Lung	2.3	2
5	60	M	Epithelioid	T3N1M0	EPP, chemotherapy, ICI, Radiation	Chest wall	1.8	2
6	74	M	Epithelioid	T3N0M0	Chemotherapy, ICI, Radiation	Lung	1.5	1
7	77	M	Epithelioid	T3N0M0	P/D, chemotherapy	Lung	2.2	1
8	69	M	Biphasic	T1N0M0	P/D, ICI	Lung Chest wall	2.1	6
9	65	M	Epithelioid	T3N0M0	P/D, chemotherapy	Chest wall	1.5	1
10	69	M	Epithelioid	T1bN0M0	P/D, chemotherapy	Chest wall	1.3	2
11	67	F	Epithelioid	T1N0M0	P/D, chemotherapy	Lung	1.3	1
12	68	M	Epithelioid	T2N1M0	P/D, chemotherapy	Lung	2.2	1
13	74	M	Epithelioid	T1N0M0	P/D, ICI	Chest wall	1.3	2
14	72	M	Epithelioid	T2N0M0	P/D, chemotherapy, ICI, Radiation	Chest wall	1.8	1

## Data Availability

The data presented in this study are available upon request from the corresponding author. The data are not publicly available due to privacy and ethical restrictions.

## Abbreviations

The following abbreviations are used in this manuscript:

RFA	Radiofrequency ablation
PM	Pleural mesothelioma
ICI	Immune checkpoint inhibitor
IMRT	Intensity-Modulated Radiotherapy
CT	Computed Tomography
EPP	Extrapleural pneumonectomy
P/D	Pleurectomy/Decortication
RT	Radiotherapy
FDG-PET/CT	Fluorodeoxyglucose Positron Emission Tomography/Computed Tomography
AE	Adverse event
